# Cognitive function in patients undergoing cystectomy for bladder cancer – results from a prospective observational study

**DOI:** 10.1177/17562872221087660

**Published:** 2022-03-24

**Authors:** Camilla M. Grunewald, Vera Feldmeier, Tillmann Supprian, Peter Albers, Markus Giessing, Günter Niegisch

**Affiliations:** Department of Urology, Medical Faculty, Heinrich Heine University, Moorenstr. 5, 40225 Duesseldorf, Germany; Department of Urology, Medical Faculty, Heinrich Heine University Düsseldorf, Düsseldorf, Germany; Department of Psychiatry and Psychotherapy, Medical Faculty, Heinrich Heine University Düsseldorf, Düsseldorf, Germany; Department of Urology, Medical Faculty, Heinrich Heine University Düsseldorf, Düsseldorf, Germany; Division of Personalized Early Detection of Prostate Cancer, German Cancer Research Center (DKFZ), Heidelberg, Germany; Department of Urology, Medical Faculty, Heinrich Heine University Düsseldorf, Düsseldorf, Germany; Department of Urology, Medical Faculty, Heinrich Heine University Düsseldorf, Düsseldorf, Germany

**Keywords:** bladder cancer, mild cognitive impairment, radical cystectomy

## Abstract

**Background:**

Impaired cognitive function of bladder cancer patients plays a role in coping with the kind of urinary diversion and may impact perioperative morbidity. In this study we therefore aimed to assess the prevalence of mild cognitive impairment in patients undergoing radical cystectomy. Secondary objectives included correlation of common cognition tests, assessment of the admitting physician, and perioperative complication rates.

**Methods:**

Patients undergoing radical cystectomy for bladder cancer were prospectively screened by neuropsychological tests including cognition tests [DemTect (Dementia Detection test), MMSE (Mini-Mental State Examination), clock drawing test] prior to surgery. Besides, clinical characteristics and perioperative outcomes were documented. Frequency of mild cognitive impairment as assessed by DemTect was correlated with the results of MMSE and clock drawing test, the occurrence of anxiety and depression, the assessment of the admitting physician, and perioperative complication rates as calculated by Spearman rank correlation coefficient. Comparative analysis (parametric and nonparametric) of patient characteristics (nonpathological *versus* pathological DemTect suggestive of mild cognitive impairment) was performed.

**Results:**

A total of 51 patients (80% male, median age 69 years) were analyzed. DemTect was suspicious of mild cognitive impairment in 27% (14/51) of patients, whereas MMSE and clock drawing test showed pathological results only in 10/51 and 6/51 patients, respectively. We found no correlation between mild cognitive impairment and anxiety/depression status. In all, 5/20 patients (25%) with suspicious DemTect results were considered suitable for a continent diversion neobladder by the admitting physician. Suspicious DemTect results were predictive for higher perioperative complication rates (29% *versus* 5%). Study limitations include small sample size and missing long-term follow-up.

**Conclusions:**

Mild cognitive impairment was observed in more than a quarter of radical cystectomy patients prior to surgery. Preoperative assessment should be supplemented by neuropsychological testing such as the DemTect as mild cognitive impairment is often underestimated and associated with significantly higher perioperative complication rates.

## Introduction

Bladder cancer (BC) is a frequent solid malignancy with 25% of the patients presenting muscle-invasive disease (MIBC) at first diagnosis.^1^ Treatment of choice in patients with nonmetastatic MIBC is radical cystectomy (RC) associated with a considerable risk of high-grade complications (13–40%)^2,3^ with open and robotic surgery showing comparable complication rates^4,5^ but beneficial results with regard to quality of life for robotic surgery.^6^

The most common urinary diversions after RC include the incontinent ileal conduit (IC) and the more complex but continent ileal neobladder (INB) with each one influencing quality of life to different degrees while bearing different risks for complications.^7^

Mild cognitive impairment (MCI) is a transitional state between normal, age-associated cognitive decline and dementia. In Germany, the prevalence among persons aged 66–80 is 9–14%.^8^ Simple psychometric test procedures such as the Dementia Detection Test (DemTect), the Mini-Mental State Examination (MMSE), or the clock drawing test allow the orienting clarification when cognitive impairment is suspected.^9–11^ Since, in addition to neurodegenerative brain changes, anxiety and depression also lead to sometimes considerable impairment of cognitive functions, careful diagnostic differentiation is essential.^12^

Especially, for potential neobladder recipients, correct assessment of their suitability for this kind of diversion is of utmost importance. Besides physical limitations including chronic inflammatory bowel disease, renal insufficiency, or tumor-specific factors, patients scheduled for neobladder must have the cognitive abilities to learn how to handle a neobladder correctly and to recognize potential malfunctions in time.^13–15^ As currently the decision on urinary diversion depends rather on the subjective assessment of the patient’s cognitive abilities as on the use of validated instruments, we aimed to evaluate the prevalence of MCI in this population by using a standardized test battery. Furthermore, patients’ individual appraisal of cognitive functioning and assessment of the admitting physician regarding suitability for a neobladder was assessed as was perioperative complication rate depending on DemTect test result.

## Materials and methods

### Study design and patients

In this prospective observational study, between March 2011 and November 2014, patients scheduled for RC underwent a neuropsychological examination following written consent up to 2 days before surgery. Prior to the examination, all patients were reviewed for their study suitability. Inclusion criteria were age ⩾18 years, the ability to consent, and a histologically confirmed malignant tumor of the bladder. Exclusion criteria were known cognitive deficits (dementia, mental retardation), drug abuse (except tobacco smoking), or other previous psychiatric illnesses (e.g. severe depression).

The study was approved by the Ethics Committee of Heinrich Heine University Düsseldorf, reference number 3606, and conducted in accordance with the principles of the Helsinki Declaration and the recommendations of good clinical practice (GCP).

### Assessments

The neuropsychological test battery included both cognitive tests (DemTect, MMSE, clock drawing test)^9–11^ and tests for distress/anxiety [HADS-D(Hospital Anxiety and Depression Scale, german version), NCCN (National Comprehensive Cancer Network) distress thermometer].^16,17^ The tests were performed by two MD students, who underwent an according training within a multiday internship in the memory clinic at the Department of Psychiatry and Psychotherapy of the Heinrich-Heine-University, Düsseldorf, Germany.

The DemTect comprises five short tasks examining a range of cognitive functions (e.g. perception, learning, memory, reasoning). It summarizes to a maximum score of 18 points with ⩾ 13 points being the cut-off value for normal cognitive function. Scores ⩽ 12 point toward cognitive impairment (scores 9–12 indicate MCI, scores ⩽ 8 suggest dementia) and are thus regarded as pathological.^9^

Similarly, the MMSE is used to test central cognitive functions on the basis of 11 tasks. For the MMSE screening test, normal cognitive function is assumed when reaching scores ⩾ 28.^10^

For the clock drawing test, the patient is asked to draw the digits of a clock in a circle and to draw the clock hands so that they represent a given time. Depending on the deviation to normal performance, 1 to 6 points are assigned and a score of ⩾ 3 is considered as an indicator for dementia.^11^

The HADS-D questionnaire contains seven items each referring to either depression or anxiety. For each subscale the patient can achieve a maximum of 21 points. Values ⩾ 11 are regarded as certainly pathological and  ⩽ 7 as unremarkable. Scores from 8 to 10 are considered borderline.^16^

The NCCN distress thermometer consists of a visual scale in the form of a thermometer ranging from 0 (not at all stressed) to 10 (extremely stressed) and a consecutive problem list. A cut-off value of 5 is regarded as an indicator for significant distress.^17^

All patients meeting the inclusion criteria were screened accordingly. In addition to the neuropsychological examination, there was a detailed documentation of family as well as social and medical history. Comorbidities were documented and graded using the Charlson Comorbidity Index (CCI)^18^ and performance status was evaluated according to the recommendations of the Eastern Cooperative Oncology Group (ECOG).^19^ Moreover, the American Society of Anesthesiologists (ASA) risk classification^20^ as well as the Barthel index^21^ were used for further patient assessment. Patients’ quality of life was assessed using the German version of the QLQ-C30 of the EORTC (European Organisation for Research and Treatment of Cancer, https://www.eortc.be).^22^ Beyond, the admitting physician had to assign points ranging from 1 (not at all suitable) to 10 (most suitable) with a score >5 being the cut-off for general neobladder suitability. This evaluation was performed independently and without knowledge of the cognitive test results.

### Outcomes

Primary endpoint of this trial was to assess the prevalence of MCI in patients scheduled to undergo RC for invasive BC as assessed by the DemTect. Secondary endpoints included the correlation of DemTect results to MMSE and clock drawing test results as well as the comparison of patients with pathological and nonpathological DemTect results regarding perioperative complications and baseline characteristics.

### Statistical analyses

With regard to the primary endpoint, assuming that the prevalence of MCI in the age-matched normal population was approximately 10% at a given significance level of alpha = 5%,^8^ the study was designed to detect a prevalence of MCI of less than 20% with a power of 80% (ß = 20%) in cystectomy patients. To test the hypothesis, a one-sided test was used requiring a case number of approximately 56 patients. Of 56 patients tested, at least 11 would need to have a pathological DemTect to deny the null hypothesis.

Results of descriptive analysis were presented as median with interquartile range for continuous variables and as frequencies with percentages for categorical variables.

Mann–Whitney *U* and Fisher’s exact tests were used for according comparison of groups. A *p* value of  < 0.05 was considered statistically significant. To calculate statistical relationships of different variables, the Spearman rank correlation coefficient was used. Propensity score analysis was performed to further evaluate the impact of DemTect results on perioperative complications. Propensity score was calculated including age, smoking habits, body mass index, arterial hypertension, diabetes, type of surgery (open *versus* robotic cystectomy), type of urinary diversion, tumor stage, Barthel index, ASA, ECOG, and CCI. Outcome was tested using logistic regression analysis.

## Results

### Study population and baseline characteristics

Between March 2011 and November 2014, 56 patients undergoing RC for BC were included in this prospective trial. Of these, 51 patients were included in the analysis regarding the primary endpoint. Five patients withdrew their consent either following or before their cognitive testing for unknown reasons. Baseline characteristics of the 51 patients analyzed are detailed in Table 1.

**Table 1.** Baseline characteristics of patients included in the analysis according to nonpathological and pathological DemTect results.

	All patients (*n* = 51)	Nonpathological DemTect (*n* = 37)	Pathological Dem Tect (*n* = 14)	*p* value
Age, median (IQR)	69 [62–75]	66 [59–73]	75 [67–78]	0.060
Gender, (*n* (%)				1.000
Female	10 [20]	7 [19]	3 [21]	
Male	41 [80]	30 [81]	11 [79]	
Barthel index, median (IQR)	100 [95–100]	100 [95–100]	98 [91–100]	0.461
ECOG PS, median (IQR)	1 [0–1]	0 [0–1]	1 [1–2]	**0.006**
ASA score, median (IQR)	2 [2–3]	2 [2–3]	3 [2–3]	0.501
CCI, median (IQR)	3 [2–4]	3 [2–3]	5 [3–6]	**0.010**
Smoking history, *n* (%)	29 [57]	22 [59]	7 [50]	0.752
Arterial hypertension, *n* (%)	20 [39]	15 [41]	5 [36]	1.000
BMI, median (IQR)	25 [23–29]	25 [23–29]	27 [24–29]	0.657
Diabetes, *n* (%)	9 [18]	5 [14]	4 [29]	0.236
pT, *n* (%)				0.120
<T2	15 [29]	14 [38]	1 [7]	
T2	17 [33]	12 [32]	5 [35]	
T3	9 [18]	5 [14]	4 [29]	
T4	10 [20]	6 [16]	4 [29]	
pN, *n* (%)				0.468
0	35 [69]	27 [73]	8 [57]	
+	12 [23]	7 [19]	5 [36]	
x	4 [8]	3 [8]	1 [7]	
Surgical margins, *n* (%)				0.419
Negative	43 [84]	30 [81]	13 [93]	
Positive	8 [16]	7 [19]	1 [7]	
Surgery type, *n* (%)				0.309
Open radical cystectomy	13 [25]	8 [22]	5 [36]	
Robotic cystectomy	38 [75]	29 [78]	9 [64]	
Diversion type, *n* (%)				0.023
Ileal conduit	29 [57]	17 [46]	12 [86]	
Neobladder	16 [31]	15 [40]	1 [7]	
Other	6 [12]	5 [14]	1 [7]	

ASA, American Society of Anaesthesiologists; BMI, body mass index; CCI, Charlson Comorbidity Index; ECOG, Eastern Cooperative Oncology Group performance status; IQR, interquartile range.

Statistical analysis was performed using Fisher’s exact test for categorical and Mann–Whitney *U* test for continuous variables.

ECOG and CCI scores were significantly higher in patients with MCI according to DemTect results being referred to as patients with pathological DemTect (*p* = 0.006 and *p* = 0.010). In total, 22% of the patients were smokers in the nonpathological DemTect group compared with only 7% smokers in the pathological DemTect group without statistical significance (*p* = 0.752). Comparably more patients in the pathological DemTect group suffered from diabetes (*p* = 0.236). Also, in the pathological DemTect group, more advanced tumors were found overall, whereas more patients in the nonpathological DemTect group received robotic cystectomy compared with open cystectomy (*p* = 0.309).

### Cognitive tests

**Figure 1.**
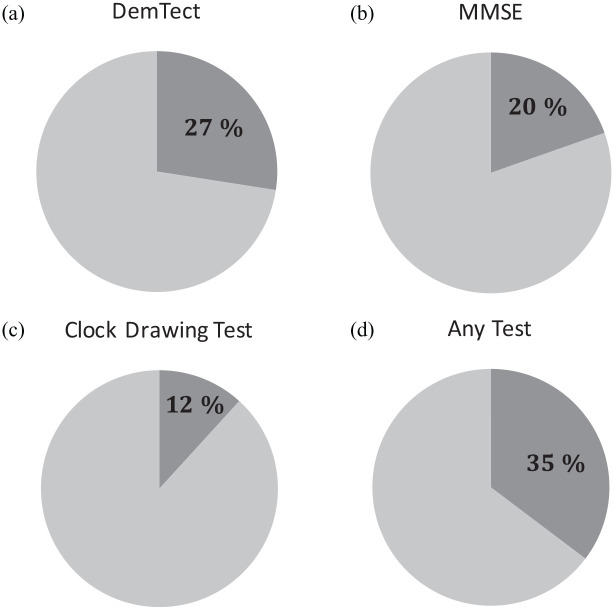
Frequency of MCI findings in (a) DemTect, (b) MMSE, (c) clock test, or (d) any of the three tests.

Within our study population, median DemTect score was 14 [interquartile range (IQR): 12–17]. As depicted in Figure 1(a), we found pathological DemTect results, that is, at least beginning cognitive impairment in 14/51 patients. With 27%, the prevalence of cognitive impairment was significantly higher within our study population compared with the normal population according to our statistic assumptions (see the ‘Materials and Methods’ section for further information).

In parallel, we performed MMSE with all patients. Median MMSE score in our population was 29 (IQR: 28–30). Only 10/51 patients (20%) showed impaired cognitive function according to this test (Figure 1(b)). Correlation analysis showed statistically significant strong correlation between DemTect and MMSE results (*r_s_* = 0.59, *p* < 0.001, Table 2).

**Table 2.** Correlation between DemTect test results and MMSE, clock drawing test, HADS-D, NCCN distress thermometer, EORTC QLQ-C30 (item 20/25) test results, or medical opinion as calculated using Spearman rank correlation coefficient (*r_s_*) with *p* < 0.05 being considered as statistically significant.

	DemTect	
	*r_s_*	*p* value
MMSE	0.59	<0.001
Clock drawing test	0.296	0.035
HADS-D (anxiety)	–0.089	0.593
HADS-D (depression)	–0.254	0.124
NCCN distress thermometer	–0.010	0.948
EORTC QLQ-C30 (item 20/25)	0.341	0.029
Medical opinion	0.149	0.424

In addition to DemTect and MMSE, we also performed clock drawing test with all patients. Median score here was 8 (IQR 8–10). Only 6/51 patients (12%) had ⩽ 5 points suggesting cognitive impairment in those patients according to this test modality^11^ (Figure 1(c)). Correlation analysis again showed statistically significant albeit only moderate correlation between DemTect and clock drawing test results (*r_s_* = 0.296, *p* = 0.035, Table 2).

Overall, 18/51 patients, that is, 35%, showed signs of cognitive impairment in any of the three tests (Figure 1(d)) with DemTect being the most sensitive one.

### Anxiety and depression

Only 38/51 patients (75%) agreed to answer questions regarding their mental health using the HADS-D questionnaire. Of those, 11 patients (29%) had shown signs of MCI according to DemTect results.

According to HADS-D results, 8% of patients (3/38) were shown to suffer depressive symptoms and 21% (8/38) had significant signs of anxiety. More than one-third of the patients (34%) had borderline signs of depression, and almost one-third (29%) had borderline or definite anxiety. About two-thirds of the patients were unaffected.

**Figure 2.**
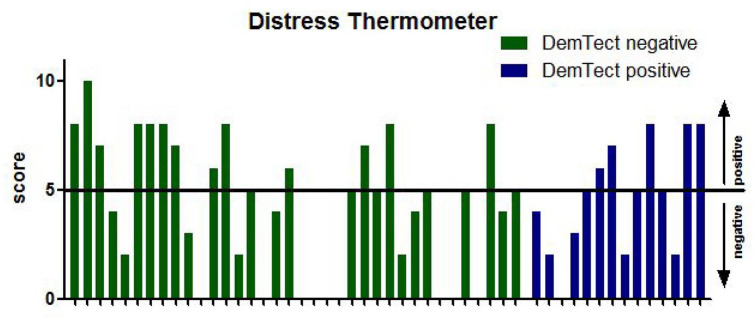
Results of the distress thermometer depicted as a score from 0 to 10 on the *y*-axis in patients with nonpathological (green bars) and pathological DemTect results (blue bars) with the horizontal line representing the cut-off value of 5. In case of missing bars, the according patient refused to fill in the distress thermometer form.

In the same way, patients were asked to answer the NCCN distress thermometer as a measurement of psychosocial burden.^17^ Here, 44/51 patients (86%) agreed to participate. Of those, 13 patients (30%) had shown signs of cognitive impairment following DemTect results. As depicted in Figure 2, 18 of the 44 tested patients (41%) showed suspicious test results.

Importantly, following subsequent correlation analysis, no correlation was seen between DemTect and HADS-D or NCCN distress thermometer results (*r_s_* = −0.089 (anxiety), *r_s_* = −0.254 (depression) and *r_s_* = −0.010, respectively, Table 2).

### Patients’ awareness

In total, 41/51 patients (80%) answered the EORTC QLQ-C30 questionnaire. As we were especially interested in patients’ own awareness of their cognitive status, we focused on questions aiming at cognitive functions (item numbers 20 and 25).^22^ There was a moderate correlation between DemTect results and patients’ awareness (*r_s_* = 0.341, *p* = 0.029, Table 2). Only 2/14 patients (14%) with suspicious DemTect results complained about impaired cognitive function as reported by EORTC QLQ-C30.

### Correlation medical opinion – DemTect results

Especially, for potential neobladder recipients correct assessment of their suitability for this complex urinary diversion is of utmost importance. We therefore compared subjective assessment by the admitting physician to objective DemTect results. In total, 31/51 patients (61%) were assessed by the admitting physician accordingly.

Only very weak correlation with DemTect results was seen for these patients (Table 2, *r_s_* = 0.149, *p* = 0.424). Importantly, 5/20 patients (25%) considered suitable for a neobladder by the admitting physician showed pathological DemTect results. In the end, fortunately, as depicted in Table 1, only one patient received a neobladder within the pathological DemTect group.

### Perioperative complications according to Clavien–Dindo

To further analyze the effects of pathological DemTect results on perioperative complications, we performed univariate analysis comparing patients with and without perioperative high-grade complications as defined by Clavien–Dindo grade ⩾4. In fact, patients with MCI according to preoperative DemTect results significantly more often experienced high-grade complications, both in dichotomous and continuous analysis (*p* = 0.042 and *p* = 0.0238, respectively, Table 3). Of the 14 patients with MCI according to DemTect results, 4 (29%) showed life-threatening complications compared with only 2/37 patients (5%) with unremarkable DemTect results. Within the pathological DemTect group those severe complications included one intestinal anastomotic and one conduit insufficiency, one severe secondary hemorrhage with subsequent sepsis, and one myocardial infarction (with fatal outcome in two cases). The two patients with nonpathological DemTect results both suffered from intestinal anastomotic insufficiency (no deaths). Interestingly, no other variable reached statistical significance in this analysis (Table 3). Nevertheless, to account for possible confounders in our analysis we performed subsequent propensity score analysis as outlined in the section ‘Materials and Methods’. After adjusting for propensity score, patients with pathological DemTect results were still shown to be at higher risk of high-grade complications (*p* = 0.034).

**Table 3.** Univariate analysis of the effect of clinical baseline characteristics and DemTect results on perioperative complication rates.

	All patients (*n* = 51)	CDC I–III (*n* = 33)	CDC ⩾ IVa (*n* = 6)	*p* value
Age, median (IQR)	69 [62–75]	69 [63–76]	68 [61–68]	*p* = 0.888
Gender, *n* (%)				*p* = 0.290
Female	10 [20]	5 [15]	2 [33]	
Male	41 [80]	28 [85]	4 [67]	
Barthel index, median (IQR)	100 [95–100]	100 [90–100]	100 [95–100]	*p* = 0.984
ECOG PS, median (IQR)	1 [0–1]	1 [0–1]	1 [0–1]	*p* = 0.968
ASA score, median (IQR)	2 [2–3]	2 [2–3]	3 [2–3]	*p* = 0.156
CCI, median (IQR)	3 [2–4]	3 [2–4]	5 [3–5]	*p* = 0.549
DemTect, median (IQR)	14 [12–17]	16 [13–17]	12 [11–12]	***p* = 0.0238**
DemTect, *n* (%)				***p* = 0.042**
Nonpathological	37 [73]	26 [79]	2 [33]	
Pathological	14 [27]	7 [21]	4 [67]	
Smoking History (*n*(%)	29 [57]	19 [58]	3 [50]	*p* = 1
Arterial Hypertension (*n*(%)	20 [39]	12 [36]	3 [50]	*p* = 0.658
BMI, median (IQR)	25 [23–29]	25 [23–27]	27 [26–33]	*p* = 0.101
Diabetes, *n* (%)	9 [18]	3 [9]	2 [33]	*p* = 0.161
pT, *n* (%)				*p* = 0.293
<T2	15 [29]	9 [27]	1 [17]	
T2	17 [33]	14 [42]	3 [50]	
T3	9 [18]	3 [9]	2 [33]	
T4	10 [20]	7 [21]	0 [0]	
pN, *n* (%)				*p* = 0.171
0	35 [69]	25 [76]	4 [66]	
+	12 [23]	8 [24]	1 [17]	
x	4 [8]	0 [0]	1 [17]	
Surgical margins, *n* (%)				*p* = 1
Negative	43 [84]	28 [85]	5 [83]	
Positive	8 [16]	5 [15]	1 [17]	
Surgery type, *n* (%)				
Open radical cystectomy	13 [25]	8 [24]	3 [50]	*p* = 0.323
Robotic cystectomy	38 [75]	25 [76]	3 [50]	
Diversion type, *n* (%)				*p* = 0.493
Ileal conduit	29 [57]	16 [49]	5 [83]	
Neobladder	16 [31]	14 [42]	1 [17]	
Other	6 [12]	3 [9]	0 [0]	

ASA, American Society of Anaesthesiologists; BMI, body mass index; CCI, Charlson Comorbidity Index; CDC, Clavien–Dindo classification; ECOG PS, Eastern Cooperative Oncology Group performance status; IQR, interquartile range.

Statistical analysis was performed using Fisher`s exact test for categorical and Mann–Whitney *U* test for continuous variables.

## Discussion

Due to improved perioperative management, an increasing number of elderly and multimorbid patients are nowadays being treated by RC, resulting in an increase in patients with cognitive deficits in this population. As these are often only slight impairments not affecting activities of daily living, so-called MCI, they often remain undetected.

There are only little data on the prevalence of cognitive deficits in BC patients, although this is especially important in the light of urinary diversion and perioperative complications. One study published in 2016 examined 90 patients by MMSE before and after cystectomy with 7.8% of patients showing signs of impaired cognition preoperatively. The majority of patients presented a postoperative stable MMSE outcome, although pre-existing deficits were found to be the greatest risk factor for postoperative deterioration.^23^ Similarly, a study from 2013 already showed that preoperatively lower MMSE scores were associated with a higher probability of developing postoperative delirium and the need for surgical revision in patients undergoing RC, although no assumptions on MCI prevalence can be drawn from this trial.^24^

Besides determining the prevalence of MCI in patients with MIBC before RC and identifying possible risk factors, the aims of this study included comparison of DemTect, MMST, and clock drawing test results as well as differentiation of MCI from anxiety and depression and to examine the relationship between MCI and perioperative complication rates.

Overall, the patient population of this study was similar to other published BC collectives, although a comparatively low proportion of patients with locally advanced tumor stages were included (pT3: 18% *versus* 31.5% and 36%, respectively).^3,25^

Unlike the work of Schoenenberger *et al.* which relied on MMSE results alone, we used DemTect, MMSE, and clock drawing test. While the reported sensitivity of MMSE and clock drawing test is 62% and 85%, respectively,^11,26^ the sensitivity of DemTect is reported to be 80%^9^ with significantly higher specificity (71.7% *versus* 45.6%) compared with the clock drawing test.^9,27^ In this study, we were able to identify the highest proportion of patients with MCI using DemTect, although assumptions regarding sensitivity and specificity can hardly be made. Using the DemTect test, 14/51 patients (27.5%) showed signs of MCI, a statistically significant higher proportion of patients compared with the assumed 10% prevalence of MCI in the age-matched normal population reported by Dlugaj *et al.*^8^ One reason for the increased incidence of MCI in the cystectomy patients included in this study may be the large proportion of smokers or former smokers in this study (57%). Tobacco smoking is considered to be one of the most important risk factors for the development of both BC and dementia. However, there was no statistically significant difference of smokers between the nonpathological and pathological DemTect group. In fact, with regard to considered risk factors for dementia, we found a corresponding trend only in relation to diabetes, however without statistical significance. Generally, patients with MCI according to DemTect results showed a statistically significant higher ECOG as well as CCI (*p* = 0.006 and *p* = 0.010, respectively) which is a surrogate that patients were generally older and sicker in this group, although age could be excluded as the sole risk factor (*p* = 0.060). Importantly, even when evaluating the results of MMSE and clock drawing test, the MCI prevalence was significantly higher than that of the age-matched normal population from Dlugaj *et al.*^8^ Correlation analysis further confirmed statistical significant correlation between DemTect and MMSE or clock drawing test results, respectively (*p* < 0.001 and *p* = 0.035).

Cognitive impairment is a common symptom of depressive disorders^28^ with 45% of patients suffering a depression prior to cystectomy.^29^ In order not to overlook a possible negative influence of preoperative anxiety and emotion on testing, we performed additional tests with regard to these factors (HADS-D for fear and depression and NCCN distress thermometer for psychosocial burden). DemTect scores showed no correlation with HADS-D test scores or distress thermometer scores, so neither depressed mood nor patient anxiety appeared to have had an impact on cognitive performance in this patient population. However, consistent with the results of a large meta-analysis,^30^ a moderate association between DemTect results and patients’ awareness (EORTC QLQ-C30 items 20 + 25) was demonstrated, suggesting that patients may have already been aware of their cognitive deficits. Small sample size obviously reduces reliability of this observation.

The neuropsychological testing performed in this study is usually not implemented in the clinical routine when patients are admitted to hospital for RC. Normally, it is a shared decision-making between the treating physician and the patient to decide on the type of urinary diversion. In this context, it seems concerning that very little correlation was found between DemTect outcomes and medical opinion with respect to the form of urinary diversion (*r_s_* = 0.149, *p* = 0.424). However, this can be easily explained by patients who showed good DemTect results but were judged unsuitable for a neobladder by the admitting physician. Obviously, parameters such as physical disabilities, previous bowel surgery, and tumor-specific factors play a role in this context as well. Although only one patient of the pathological DemTect group received a neobladder in the end, it is an important finding of our study that 5/20 patients (25%) initially considered suitable for a neobladder by the admitting physician showed pathological DemTect results. Together with the fact that patients with MCI according to DemTect results showed significantly higher complication rates according to Clavien–Dindo compared with patients with nonpathological DemTect results (29% *versus* 5% life-threatening complications, respectively, *p* = 0.042), we come to the conclusion that preoperative assessment of patients undergoing RC should be supplemented by simple cognition testing such as the DemTect. Limitations of our analysis include small sample size, missing long-term follow-up, and the comparatively old data, although BC population has not changed significantly over the last years.

Prevalence of MCI is significantly higher in the RC population than in the age-matched normal population and often underestimated by the treating physician. Whether the use of the DemTect is sufficient on its own to detect MCI or whether it should be supplemented with other testing modalities, as well as the extent to which the presence of MCI influences the long-term outcome of patients undergoing RC, is left for future investigation.

## Supplemental Material

sj-doc-1-tau-10.1177_17562872221087660 – Supplemental material for Cognitive function in patients undergoing cystectomy for bladder cancer – results from a prospective observational study Click here for additional data file. Supplemental material, sj-doc-1-tau-10.1177_17562872221087660 for Cognitive function in patients undergoing cystectomy for bladder cancer – results from a prospective observational study by Camilla M. Grunewald, Vera Feldmeier, Tillmann Supprian, Peter Albers, Markus Giessing and Günter Niegisch in Therapeutic Advances in Urology
